# Prevalence and pattern of skin disorders among children living in orphanages in Fayoum and Giza governorates, Egypt

**DOI:** 10.1186/s41182-025-00685-w

**Published:** 2025-01-20

**Authors:** Noha Ezzat Mohamed, Wafaa Yousif Abdel-Wahed, Shimaa Mohammed Gomaa, Mai Ashraf Hosny

**Affiliations:** 1 Faculty of Medicine, Fayoum University, Fayoum, Egypt; 2 Fayoum Derma and Leprosy Hospital, Fayoum, Egypt

**Keywords:** Skin disorders, Orphan children, Infectious, Noninfectious, Psycho-cutaneous

## Abstract

**Background:**

Childhood is a crucial period that shapes a person’s growth and development. For orphans, a lack of familial support affects their upbringing, making orphanages crucial for care. Children living in orphanage centers are vulnerable to several conditions, including dermatological disorders, due to factors such as malnutrition, overcrowding, and poor hygiene. The current study was carried to determine the prevalence and patterns of skin disorders among orphan children in Egypt, and to identify associated risk factors among orphans in Egypt.

**Methods:**

A cross-sectional study was conducted among 415 children aged 1–18 years living in orphanage centers in Fayoum and Giza governorates, Egypt.

**Results:**

Among the children, 77.1% had at least one skin disorder, with noninfectious conditions being more prevalent than infectious conditions (67.4 vs. 24.3%). The most common noninfectious conditions were dermatitis/eczema, pigmentary disorders, skin appendage disorders, and psycho-cutaneous issues. Fungal infections were the most common infectious condition. There was a significant relationship between hygiene and the type of skin disorder.

**Conclusion:**

The prevalence of skin diseases in orphanages in Egypt is relatively high and is dominated by noninfectious skin diseases, and the prevalence of psycho-cutaneous disorders is high among these children. Comprehensive care strategies focusing on improving hygiene and emotional support and preventing abuse are essential for reducing the incidence of skin disorders and enhancing the overall health of orphaned children.

## Introduction

Childhood is a critical period marked by growth and development, during which various biological and psychosocial factors influence health outcomes, including skin disorders [1, 2]. Skin conditions in children are influenced by factors such as diet, environmental exposure, socioeconomic status, overcrowding, and personal hygiene, especially in developing countries such as Egypt. Early detection and management of these disorders are essential for preventing complications such as scarring and chronic infections [3].

Orphaned children, defined by UNICEF as those under 18 years of age who have lost one or both parents, represent a particularly vulnerable group. Globally, an estimated 153 million children were classified as orphans in 2023, with many facing additional health challenges due to their living conditions. In Egypt, children living in orphanages often reside in environments with limited resources, overcrowding, shared facilities, and poor sanitation, all of which contribute to a high prevalence of skin disorders [4, 5].

The absence of familial support also increases the risk of psychological distress, contributing to psycho-cutaneous conditions such as neurotic excoriations, trichotillomania, and dermatitis artefacta [6, 7]. Moreover, inadequate nutrition, which is common in institutional settings, can result in deficiencies that manifest as skin changes, including pallor, roughness, and hair abnormalities [8].

Addressing the health needs of these children requires understanding the specific risk factors and prevalence of dermatological conditions in this population. There is limited information on the burden of skin disorders among orphans in Egypt. This study aims to determine the prevalence and patterns of skin disorders among orphaned children in Egypt, and to identify associated risk factors among orphans in Egypt.

## Methods

### Study design and setting

This descriptive cross-sectional study was conducted in orphanage centers in Fayoum and Giza, Egypt, from September 2022 to March 2023. In Fayoum, there are four available centers with 240 children. In Giza, there are 56 available centers with 1131 children.

### Study population

The study included 415 orphan children aged from 1to 18 years old, selected from 20 orphanage centers (4 in Fayoum and 16 randomly chosen in Giza). In Fayoum, 227 of 240 children participated (5.4% dropout), and in Giza, 188 of 239 children participated (21.3% dropout).

The required sample size was calculated via Open-Epi software, assuming a 50% proportion, 95% confidence interval, and 5% precision. To account for potential nonresponses, the target sample size was increased by 10%. In total, 415 were participants in this study.

### Study tools

Data collection was conducted through a structured questionnaire addressing factors such as sociodemogrphic factors (age, sex, education, and contact with parent) hygiene practices (e.g., frequency of bathing, use of soap, washing and ironing of clothes, bed-sharing habits). Clinical examinations assessed skin, hair, nails, oral health, and overall nutrition, as well as measurements of height and weight. Dermatological and nutritional findings were recorded. Treatable conditions were managed on-site, and referrals to Fayoum University Hospital’s dermatology department were made when further care was needed. Health education sessions were provided to the children and their caregivers, emphasizing personal hygiene, such as regular bathing, proper hair and clothing care, and hand-washing to prevent skin issues.

To assess the validity and reliability of the questionnaire used in the study, several steps were undertaken. The questionnaire was constructed after reviewing previous research related to skin disorders and associated factors to ensure comprehensive coverage of relevant variables. Content validity was ensured through expert review by dermatologists, and public health professionals, who evaluated the questionnaire for relevance, clarity, and completeness. Face validity was assessed through pilot testing with a group of fifteen orphan children and their caregivers to ensure the questions were clear and comprehensible. Criterion validity was established by comparing questionnaire responses with clinical examination findings, ensuring consistency between reported hygiene behaviors and observed skin conditions. Reliability was assessed through internal consistency using Cronbach’s alpha, which yielded a value of 0.72, indicating acceptable reliability of the questionnaire.

### Data analysis

Data were analyzed using Statistical Package for Social Sciences (SPSS version 23). Descriptive statistics (e.g., numbers, percentages, means, and standard deviations) were used to summarize both qualitative and quantitative data. Inferential statistics were applied to compare groups using the Chi-square test, with a *p*-value less than 0.05 considered statistically significant.

## Results

In this study, the participants were divided into three age groups: children under 6 years (74 participants, 17.8%), children aged 6–12 years (184 participants, 44.3%), and those aged 13–18 years (157 participants, 37.8%). With respect to sex distribution, there were 219 males (52.8%) and 190 females (47.2%). In terms of education, 77 children (16.6%) were in preschool, 173 (41.7%) were in primary school, 69 (16.6%) were in preparatory school, 80 (19.3%) were in secondary school, and 16 (3.9%) were in colleges or institutes. Christian children made up 28% of the sample (116 participants). Communication with parents varied, with 294 children (70.8%) having no parental contact and 121 (29.2%) maintaining communication with their parents.

Hygiene practices among the orphaned children were assessed, and 57.3% bathed more than twice a week, 31.6% bathed twice a week, and 11.1% bathed once a week. Nearly all the children (99.3%) used soap, whereas 0.7% did not. Additionally, 89.2% did not iron their clothes, and 91.3% did not share garments with others. A total of 90.6% of the children wore shoes regularly, 45.1% changed their garments daily, 34.9% changed clothes every other day, and 20% did so less frequently. With respect to bedding hygiene, 87% lived in orphanages that changed bed sheets twice a week, whereas 13% had bed sheets changed once a week.

**Fig. 1:**
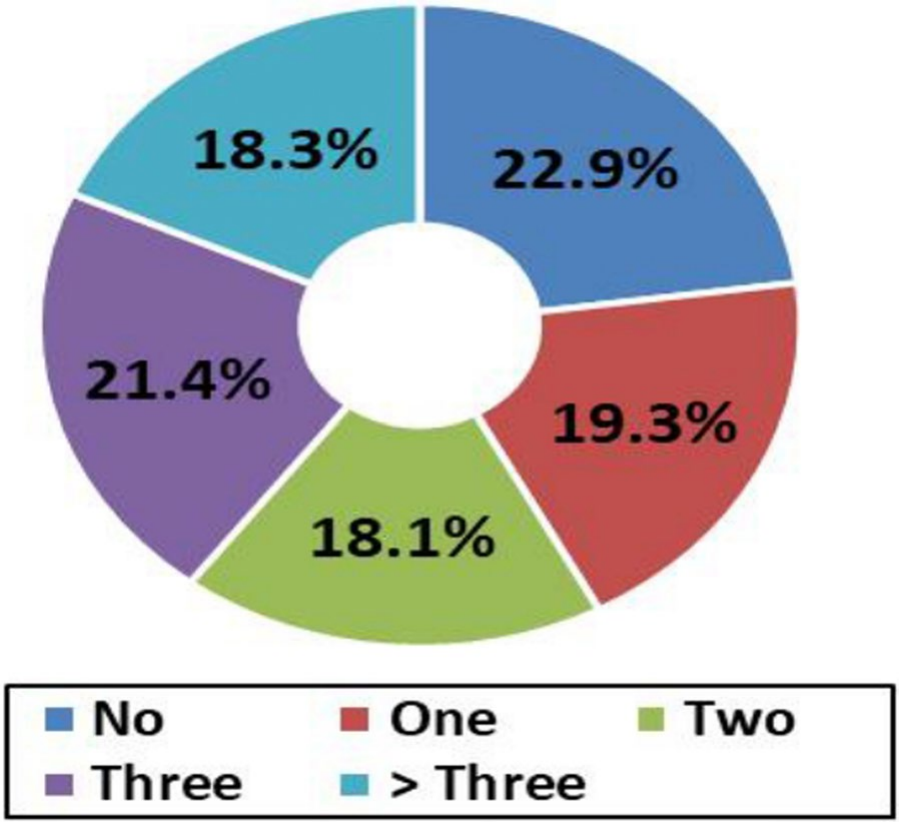
Frequency of skin disorders-among children in orphanage centers

Among the 415 children studied, about 77.1% had at least one skin lesions, about 80 (19.3%) had one dermatological disorder, 75 (18.1%) had two, 89 (21.4%) had three, and 76 (18.3%) had more than three dermatological disorders (Fig. 1). Noninfectious dermatoses were more common 288 (69.3%) than infectious dermatoses 101 (24.3%). The four most prevalent noninfectious skin conditions were dermatitis and eczema (41.9%), pigmentary disorders (27.9%), disorders of skin appendages (27%), and psycho-cutaneous disorders (26%). Among infectious skin disorders, fungal infections were the most common (10.8%), parasitic and bacterial infections represented 5.8 and 5.3%, respectively (Table 1).

**Table 1:** Prevalence of different types of infectious and noninfectious diseases

	No. 415	%
Infectious	**101**	**24.3**
Fungal	45	10.8
Bacterial	22	5.3
Parasitic	24	5.8
Viral	24	5.8
Noninfectious	**288**	**69.3**
Dermatitis and eczema	174	41.9
Papulo-squamous disorders	6	1.4
Disorders of epidermal keratinization	15	3.6
Pigmentary disorders	116	27.9
Disorders of skin appendages	112	27.0
Disorders of dermis and subcutis	43	10.4
Benign proliferation and neoplasm	39	9.4
Psycho-cutaneous disorders	108	26
Geno-dermatosis	1	0.2
Signs of physical abuse	22	5.3

Bold values indicate main category of skin disaeses

**Fig. 2:**
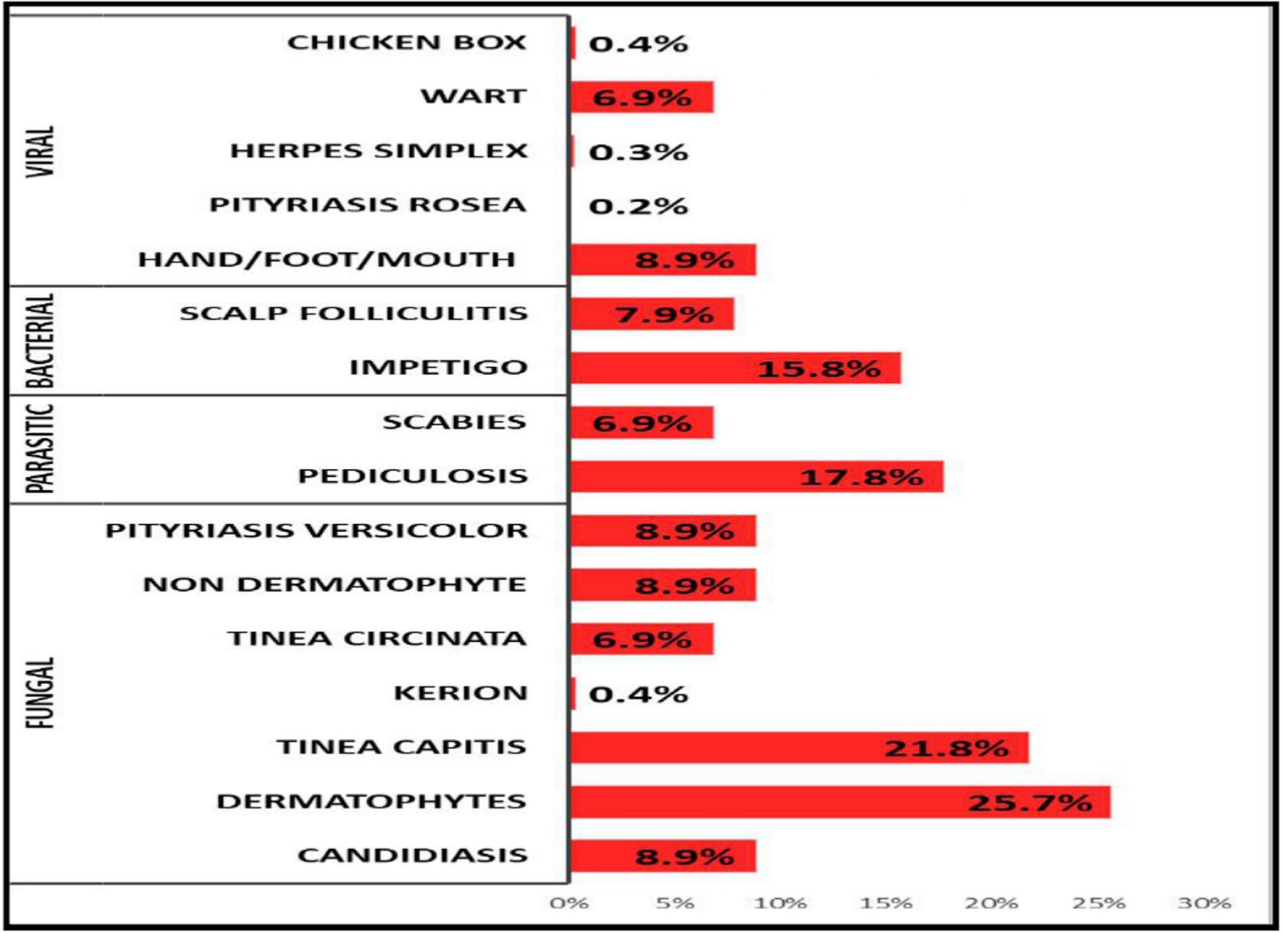
Pattern of infectious dermatological conditions among children living in orphanage centers

Among 288 children who were affected by noninfectious dermatoses, Pityriasis alba was the most common, affecting 103 children (35.8%), followed by postinflammatory hypermelanosis in 76 children (26.4%), acne in 62 (21.5%), and nail biting in 62 (21.5%). Atopic dermatitis was present in 47 children (16.3%) (Table 2). Among the 101 children with infectious dermatoses, fungal infections were the most prevalent, accounting for 43.5% of the cases, with tinea capitis being the most common type (21.8%). Pediculosis affected 17.8% of the patients, and impetigo accounted for 15.8% (Fig. 2).

**Table 2:** Pattern of noninfectious dermatological conditions among children living in orphanage centers

	Non-infectious	*N* (=288)	%
Dermatitis and eczema	Pityriasis alba	103	35.8
	Atopic dermatitis	47	16.3
	Papular urticarial	37	12.8
	Seborrheic dermatitis	25	8.7
	Hand and foot eczema	19	0.6
Papulo-squamous disorders	Psoriasis	1	0.3
	Lichen nitidus	3	1
	Lichen planus	2	0.7
Disorders of epidermal keratinization	Acanthosis nigricans	8	2.8
	Keratosis pilaris	9	3.1
Pigmentary disorders	Post-inflammatory hyper melanosis	76	26.4
	Freckles	15	5.2
	Post inflammatory hypo melanosis	13	5.4
	Vitiligo	1	0.3
	Progressive macular hypo melanosis	5	1.7
Disorders of skin appendages	Scarring alopecia	18	6.3
	Alopecia areata	15	5.2
	Traction alopecia	23	8
	Premature graying of hair	2	.7
	Telogen effluvium	9	3.1
	Acne	62	21.5
	Acneiform eruption	2	.7
	Hyperhidrosis	7	2.4
	Miliaria rubra	6	2.1
Disorders of dermis and sub-cutis	Atrophic skin disorder	24	8.3
	Hypertrophic skin disorder	15	5.2
	Keloid	3	1
	Stretchmarks	2	0.7
	Knuckle pads	7	2.4
Benign proliferation and neoplasm	Melanocytic nevus	29	10.1
	Nevus sebaceous	2	0.7
	Lipoma	2	0.7
	Milia	5	1.7
	Halo nevus	1	0.3
Psycho-cutaneous disorders	Artefactual skin disorder	7	2.4
	Trichotillomania	5	1.7
	Acne excoriee	21	7.3
	Nail biting	62	21.5
	Lip biting	30	10.4
Genodermatosis	Neurofibromatosis	1	0.3
Signs of physical abuse		22	7.6

The distributions of both infectious and noninfectious skin disorders were analyzed on the basis of factors such as age, sex, residency, parental communication, education, and religion. Infectious dermatoses were more common among younger children, particularly those aged 6–12 years, whereas noninfectious conditions increased with age and education level. Children with no communication with their parents had a higher prevalence of noninfectious skin conditions. Residency, sex and religion had no significant effect on the occurrence of skin disorders (Table 3).

**Table 3:** Distribution of skin disorders among orphan children according to demographic factors

			Infectious dermatoses		*P* value	Non-infectious dermatoses		*P* value
			Yes	No		Yes	No	
Sex	Female	N	9	47	0.766	131	65	0.284
		%	25.0%	75.0%		66.8%	33.2%	
	Male	N	52	167		157	62	
		%	23.7%	76.3%		71.7%	28.3%	
Age	0–5	N	13	61	0.02*	39	35	<0.001*
		%	17.6%	82.4%		52.7%	47.3%	
	6–12	N	60	124		122	62	
		%	32.6%	67.4%		66.3%	33.7%	
	13–18	N	43	114		127	30	
		%	27.4%	72.6%		80.9%	19.1%	
Residency	Fayoum	N	55	172	0.96	161	66	0.458
		%	24.2%	75.8%		70.9%	29.1%	
	Giza	N	46	142		127	61	
		%	24.5%	75.5%		67.6%	32.4%	
Communication with the parents	No	N	70	224	0.696	194	100	0.019*
		%	23.8%	76.2%		66.0%	34.0%	
	yes	N	31	90		94	27	
		%	25.6%	74.4%		77.7%	22.3%	
Education	Pre-school	N	24	53	0.03*	41	36	<0.001*
		%	31.2%	68.8%		53.2%	46.8%	
	Primary	N	50	123		114	59	
		%	28.9%	71.1%		65.9%	34.1%	
	Preparatory	N	13	56		57	12	
		%	18.8%	81.2%		82.6%	17.4%	
	Secondary	N	13	67		63	17	
		%	16.3%	83.8%		78.8%	21.3%	
	Faculty\institute	N	1	15		13	3	
		%	6.3%	93.8%		81.3%	18.8%	
Religion	Christian	N	21	95	0.065	84	32	0.41
		%	18.1%	81.9%		72.4%	27.6%	
	Muslim	N	80	219		204	95	
		%	26.8%	73.2%		68.2%	31.8%	

*P value <0.05, with significant difference

A significant relationship was found between hygienic factors and the occurrence of both infectious and noninfectious skin disorders, as indicated by *p* values less than 0.05 (Table 4).

**Table 4:** Distribution of noninfectious and infectious skin disorders according to hygienic factors

			Noninfectious (*n* = 288)		*P* value	Infectious (*n* = 101)		*P* value
			Yes	No		Yes	No	
Bathing frequency	Once a week	N	39	7	<0.001*	27	19	<0.001*
		%	84.8%	15.2%		58.7%	41.3%	
	Twice a week	N	114	17		37	94	
		%	87.0%	13.0%		28.2%	71.8%	
	More than twice a week	N	135	103		37	201	
		%	56.7%	43.3%		15.5%	84.5%	
Iron cloths	No	N	245	125	<0.001*	97	273	0.011*
		%	66.2%	33.8%		26.2%	73.8%	
	Yes	N	43	2		4	41	
		%	95.6%	4.4%		8.9%	91.1%	
Sharing garments	No	N	252	127	<0.001*	83	296	<0.001*
		%	66.5%	33.5%		21.9%	78.1%	
	Yes	N	36	0		18	18	
		%	100.0%	.0%		50.0%	50.0%	
Change garments	Daily	N	163	24	<0.001*	20	34	0.02
		%	87.2%	12.8%		37.0%	63.0%	
	Every other day	N	49	96		81	280	
		%	33.8%	66.2%		22.4%	77.6%	
	More than one day	N	76	7		27	19	
		%	91.6%	8.4%		58.7%	41.3%	
Change of bed sheets	Once a week	N	54	0	<0.001*	37	94	<0.001*
		%	100.0%	.0%		28.2%	71.8%	
	Twice a week	N	234	127		37	201	
		%	64.8%	35.2%		15.5%	84.5%	

*P value <0.05, with significant difference

Furthermore, psycho-cutaneous disorders such as acne excoriee, alopecia areata, nail biting, and lip biting were significantly more common among older children (above 12 years). There were notable sex differences in psycho-cutaneous disorders, with females being more affected by acne excoriee (*p* = 0.001) and males being more affected by alopecia areata (*p* = 0.012).

## Discussion

Children in orphanages are at high risk for health issues, including skin diseases. Understanding the prevalence in specific age groups is crucial for assessing healthcare needs [1]. This study is the first to explore the range of skin diseases among children in orphanages in Egypt.

The findings underscore a critical public health concern regarding the high prevalence of skin diseases among children in Egyptian orphanages, particularly in Fayoum and Giza. The 77.1% rate identified is not only significantly higher than in Egyptian schoolchildren (71.4%) [9], but also exceeds rates observed in Tanzanian orphanages (57.4%) [5]. This disparity could be influenced by various factors unique to orphanages, such as overcrowding, limited resources for hygiene, and potentially lower access to healthcare. Children in institutional settings may also experience psychosocial stress, which can impact immune function, making them more susceptible to dermatological conditions. Comparing these findings with broader Egyptian school populations and international studies reveals potential gaps in the healthcare and hygiene support provided in orphanages [7]. Low prevalence rates of dermatologic conditions were reported in more developed countries as Kuwait and Turkia [10, 11].

In our study, noninfectious skin conditions were predominant, affecting 69.3% of the children, contrasting with findings from a Tanzanian study where infectious conditions were more prevalent at 64.9% [5]. Similarly, a study in India reported a higher prevalence of infectious dermatoses at 68.5%, with noninfectious conditions affecting only 32.4% [12]. These variations may stem from environmental differences, such as climate and living conditions, which can influence the types of skin issues children encounter [13].

The most common noninfectious skin conditions in our study were Pityriasis alba (35.8%), post-inflammatory hyperpigmentation (26%), and acne vulgaris (21.5%). Similar findings were reported in Tanzanian and Indian studies [5, 12]. A research done in Sinai, Egypt has concluded that eczema or dermatitis were found in 25.8% of participants. Pityriasis alba occurred at a rate of 18.3% [11]. The high occurrence of eczema and acne in environments with poor ventilation can be attributed to multiple factors, including both excessive bathing and inadequate bathing practices. Such practices may compromise the integrity of the skin barrier, making children more susceptible to dermatological issues like atopic dermatitis. Additionally, the disruption of the skin barrier can facilitate the colonization of pathogens, particularly *Staphylococcus aureus*, which is known to exacerbate skin conditions [13].

The occurrence of Pityriasis alba among children in our study likely reflects underlying factors associated with low socioeconomic status, including inadequate vitamin intake and nutritional deficiencies [14]. Post-inflammatory hyperpigmentation may result from injuries such as insect bites or other minor traumas, particularly in settings where children are more frequently exposed to.

Infectious dermatoses affected 24.3% of participants in our study, a lower rate compared to Tanzanian and Indian studies. The most common infections were tinea capitis (21.8%), pediculosis capitis (17.8%), and impetigo (15.8%). In Sharkia, Egypt, bacterial infections, particularly impetigo, were most prevalent [15]. In Sinai Egypt, parasitic pediculosis capitis was the most prevalent skin disorder among children [9], while the Tanzanian study reported that fungal infections comprised 94% of cases, with tinea capitis (63.5%) and Pityriasis versicolor (17.7%) [5], In India, parasitic infections were most common, followed by fungal conditions [12]. The high rate of fungal infections in the current may result from inadequate hygiene, shared clothing, and humid conditions, while the prevalence of pediculosis capitis likely reflects crowded living conditions and close personal contact [13].

Our study found that infectious dermatoses were more prevalent among younger children, particularly those aged 6–12 years, while noninfectious dermatoses were more common in older children. This trend aligns with findings from other studies, which suggest that the higher rate of infectious skin conditions among younger children may stem from their increased physical interactions and close contact during play, leading to a greater risk of transmission [16].

The current study revealed the significant role of parental communication in the occurrence of skin disorders. Children who lack contact with their parents present higher rates of noninfectious skin conditions, suggesting that emotional stress or a lack of parental guidance on hygiene may be contributing factors. This aligns with existing research indicating that children in orphanages or institutionalized settings are more vulnerable to psychological stressors that manifest as skin-related issues [17].

Nutritional dermatoses were not observed in our study. This is likely because most orphan centers receive donations that help meet the children’s needs. Additionally, the majority of participants had a well-balanced diet, as indicated by normal height-to-weight ratios and the absence of clinical signs of malnutrition. However, the nutritional status observed may not fully represent the exact situation as children enter centers through varying circumstances. These findings align with a similar study conducted in Tanzania, which also reported no cases of nutritional dermatoses [5].

Psycho-cutaneous disorders accounted for 18% of noninfectious conditions, including nail biting (21.5%), lip biting (10.4%), and acne excoriee (7.3%). Behavioral and emotional deprivation in orphanages may lead to these disorders [17]. Additionally, physical abuse, including burn marks, affects 7.6% of children, likely due to maltreatment by poorly trained staff. This aligns with findings from Menoufia, Egypt, where institutionalized children often face violence from caregivers as a form of discipline [18]. This highlights the urgent need for reforms in orphanage care, including better staff training and the implementation of nonviolent, supportive caregiving practices. Creating a more nurturing environment is essential for addressing both the physical and the psychological needs of those children.

The study faced several limitations. Genital examinations were not permitted, potentially leading to underreported conditions. The inability to take photographs limited clinical documentation. Restrictions on accessing children’s personal information hindered detailed profiling, and the lack of comprehensive demographic data constrained analysis of sociodemographic risk factors. Despite these challenges, the study offered valuable insights into skin disorder prevalence among orphan children.

## Conclusion

Our study is the first of its kind in our country to study the prevalence and pattern of dermatoses among orphanage children in Egypt. The prevalence of skin diseases in orphanages in Egypt is relatively high and is dominated by noninfectious skin diseases, and the prevalence of psycho-cutaneous disorders is high among these children. A relationship between age, hygiene practices, emotional well-being, and the prevalence of skin disorders was detected. Infectious dermatoses were more common among younger children, whereas noninfectious conditions increased with age. Comprehensive care strategies that address physical hygiene, emotional well-being, and the prevention of abuse are essential for reducing the prevalence of skin disorders and improving the overall health of children.

## Data Availability

No datasets were generated or analyzed during the current study.
